# Negative Association of Interleukin-33 Plasma Levels and Schistosomiasis Infection in a Site of Polyparasitism in Rural Cameroon

**DOI:** 10.3389/fimmu.2019.02827

**Published:** 2019-12-03

**Authors:** Severin Donald Kamdem, Francis Konhawa, Erve Martial Kuemkon, Leonel Meyo Kamguia, Gladys K. Tchanana, Frungwa Nche, Alim Oumarou, Mamadou Hamza, Yasmine Ouratou, Mariette Nzoku Tcheutchoua, René Ghislain Essomba, Marie Paule Ngogang, Michel Kengne, Palmer Masumbe Netongo, Bienvenu Etogo Ondigui, Marie Claire Okomo Assoumou, Frank Brombacher, Justin Komguep Nono

**Affiliations:** ^1^Division of Immunology, Health Science Faculty, University of Cape Town, Cape Town, South Africa; ^2^Cape Town Component, International Centre for Genetic Engineering and Biotechnology, Cape Town, South Africa; ^3^Immunology of Infectious Diseases Unit, South African Medical Research Centre, Cape Town, South Africa; ^4^School of Health Sciences, Catholic University of Central Africa, Yaoundé, Cameroon; ^5^CIAB EXACT Medical Laboratory, Yaoundé, Cameroon; ^6^Faculty of Medicine and Biomedical Sciences, University of Yaoundé 1, Yaoundé, Cameroon; ^7^Ministry of Public Health, Yaoundé, Cameroon; ^8^Biotechnology Centre, University of Yaoundé 1, Yaoundé, Cameroon; ^9^National Public Health Laboratory, Ministry of Public Health, Yaoundé, Cameroon; ^10^LABOREB, Yaoundé, Cameroon; ^11^Department of Biochemistry, University of Yaoundé 1, Yaoundé, Cameroon; ^12^Wellcome Centre for Infectious Diseases Research in Africa, Institute of Infectious Diseases and Molecular Medicine (IDM), University of Cape Town, Cape Town, South Africa; ^13^The Medical Research Centre, Institute of Medical Research and Medicinal Plant Studies, Ministry of Scientific Research and Innovation, Yaoundé, Cameroon

**Keywords:** schistosomiasis, malaria, hepatitis, polyparasitism, cameroon, schoolchildren, interleukin 33, hepatic fibrosis

## Abstract

**Background:** This study aimed to investigate the association of plasma levels of IL-33, a mucosal alarmin known to elicit type-2 immunity, with infection and liver fibrosis profiles of school children from an endemic area for *Schistosoma mansoni*, malaria and hepatitis (B & C) in rural Cameroon.

**Methods:** A cross-sectional study enrolling schoolchildren from 5 public schools was conducted. Single schistosomiasis, malaria and hepatitis infections or co-infections were assessed by kato katz, microscopy, and rapid diagnostic tests, respectively. Hepatic fibrosis was assessed by ultrasound according to WHO Niamey guidelines and plasma levels of Interleukin 33 were determined by ELISA. All statistics were performed using R studio software.

**Principal findings:** We found a prevalence of 13.5% (37/275), 18.2% (50/275), and 8% (22/275), respectively for schistosomiasis, malaria and hepatitis (B or C) single infections. Only 7.6% (21/275) of co-infections were reported. Although Plasma IL-33 showed a minimal negative risk for schistosomiasis infection (AOR 0.99; 95% CI 0.97–1.01), *S. mansoni* infected participants had lower levels of plasma IL-33 (*p* = 0.003) which decreased significantly as eggs burdens increased (*p* = 0.01) with a negative Pearson coefficient of *r* = −0.22. Hepatic fibrosis occurred in 47.3% (130/275) of our study population independently from plasma levels of IL-33 (AOR 1.00; 95% CI 0.99–1.01).

**Conclusion/Significance:** Our data failed to show an association between plasma IL-33 levels and liver disease but convincingly report on a negative association between plasma IL-33 levels and schistosomiasis infection and egg burden in school children from a polyparasitic schistosomiasis endemic area.

## Introduction

Schistosomiasis is a tropical disease caused by a trematode worm of the genus *Schistosoma* which infects the host through contact with contaminated water. With around 207 million people infected worldwide, ~90% of cases occur in sub-Saharan Africa (1). Therefore, Schistosomiasis ranks as the second most important parasitic disease worldwide in terms of public health impact (2). While *Schistosoma mansoni, Schistosoma haematobium*, and *Schistosoma japonicum* are the 3 relevant species for humans (3), urogenital schistosomiasis (caused by *S. haematobium*) and intestinal schistosomiasis (caused by *S. mansoni*), in particular, are prevalent in Cameroonian adolescents and school age children (4). Besides Schistosomiasis, Malaria bears the greatest burden driven by parasitic diseases with an estimate of 216 million cases worldwide and more than 90% also occur in sub-Saharan Africa (5). In Cameroon, 30% of outpatient consultations, 24% of morbidity cases and 18.7% of mortality cases in healthcare units is attributed to Malaria (6). As malaria and Schistosomiasis infections are both endemic throughout the country, their distribution allows an overlap with other endemic diseases. In this regards, Hepatitis B and C seroprevalences were reported to be high in Cameroon rural settings (7, 8) and mixed infections were encountered with Malaria (9). Because of geographical overlap between Malaria, Hepatitis and schistosomiasis, polyinfections are common and lead into several forms of associations, aggravated health conditions, and co-morbidities (10–12). Evidence from epidemiological surveys have indicated that co-infected individuals might have increased vulnerability to other infections (13, 14) and potentially at higher risk of developing more severe disease due to interactions between the infecting pathogens (13–15). Schistosomiasis, malaria and hepatitis share some similarities in their pathogenesis particularly as the diseases they cause all affect their host liver. In fact, in the context of Malaria, upon a bite of an infected anopheles mosquito, Plasmodium sporozoite migrate toward the liver, invade the hepatocytes where they accumulate prior evasion within the circulation (16). Similarly, in the acute phase as well as in tolerant chronically infected individuals, hepatitis virus has been demonstrated to spread to the entire hepatocyte population (17) using the same receptor like Plasmodium parasite (18). Moreover, during schistosomiasis infection, it is well-documented that schistosomula migrate to the liver where they mature in adult worms to produce eggs (3, 19). Altogether, the co-occurrence of these infections could possibly impact the severity of the liver affection (20), but the dynamics of such processes are poorly known in clinical settings.

Upon infection, the interaction between the host and pathogens, involved cells and molecules which determine the course of the disease. Among them, cytokines play a vital role in the orientation of the host immune response to pathogens and ensuing pathological responses (21). In the context of schistosomiasis, particularly, many reports have now demonstrated the T helper-2 dominated host response to the infection (22, 23), which can be initiated by the alarmin IL-33 (24).

Discovered in 2005, interleukin-33 (IL-33) is a member of the IL-1 family (25) and was first described as an alarmin. However, IL-33 has been shown to induce multivalent functions, leading in anti or pro-inflammatory effects in numerous pathologies (26). While several studies focussed on the role of IL-33 in driving hepatic disease during schistosomiasis (27–29), hepatitis (30–32), or malaria (33), alone, very few investigations, if any at all, have been performed on the association of this cytokine with hepatic schistosomiasis infection and liver disease in a context of mixed infection. This study therefore aimed at investigating the association between plasma levels of IL-33, infection status and liver fibrosis profiles in school children with *S. mansoni* infection alone or associated with malaria, hepatitis B or hepatitis C.

## Materials and Methods

### Ethic Statement

Ethical approval was obtained from the Cameroon National Ethics committee for Human Health Research (Approval No. 2018/02/976/CE/NECRHH/SP) followed by authorizations from the Ministries of Basic Education and Public Health of Cameroon (631-12.18). Local authorities and schools' directors were also informed and granted us with authorizations. Assisted by school teachers, children, and legal guardians were informed on the scope of the study. Written informed consents and assents were given by children and legal guardians. All data gathered were treated anonymously by the research team. All Schoolchildren enrolled were treated with Praziquantel regardless of their parasitological status.

### Study Area and Population

The study was carried out in the Bokito subdivision, situated in the Mbam and Inoubou Division, within the Center region of Cameroon. At around 100 km north of Yaoundé the capital of Cameroon, Bokito is within a transitional zone between forest and savannah. From September to December 2018, data were collected from schoolchildren in five public schools belonging to five different villages of the endemic area namely Bongando, Ediolomo, Kedia, Yoro 1, and Yoro 2 public schools. The study took place 5 months after Mass Drug Administration (MDA) within the five sites. The protocol of treatment was based on the WHO-Tablet dose pole strategy, which estimates the number of praziquantel tablets (600 mg each) based on the participant body height and was recommended for mass-treatment of school-aged children to achieve an optimal dosage of 40 mg/kg (34, 35).

### Data Collection

Informed sessions were done by our team in presence of schoolchildren and parents or legal guardians to clearly explain the objective and the methodology of our study. After the informed sessions, each schoolchild was interviewed by a staff of our team, assisted by the legal guardian and the class teacher. All the information was recorded using a questionnaire.

### Parasitological Assay

Each schoolchild received two pre-labeled 50 ml screw-cap vials stool container and was requested to provide 2 fresh morning stool samples from 2 different day (with 5 days interval) for parasitological analyses. Two Kato-Katz smears of 41.7 mg fecal material each were prepared for each participant and microscopically examined by two independent technicians to detect and quantify *S. mansoni, S. haematobium* eggs ectopic elimination and other geohelminths as previously described (36). The participant burden was the geometric mean of intensity (GMI) from the 2 smears.

### Ultrasonography

Schoolchildren were examined using a portable ultrasonography device with convex transducer of 4 MHz. All investigations were conducted by the same clinician who was unaware of the infection status of the examined participants. Pathologic lesions were defined and recorded according to the WHO guidelines on the assessment and quantification of schistosomiasis morbidity as previously described (37). Whereas, participants with Liver Image Pattern (LIP) A or B are not likely to have periportal fibrosis (37) and were therefore considered as negative (controls) for liver disease, participants with LIP ranging from C to F were considered as *S. mansoni*-specific hepatic morbidity (38, 39). Participants with *S. mansoni* unspecific signs of hepatic morbidity (e.g., hepatitis) were still included in the study (39).

### Blood Collection and Assays

Whole blood (4 ml) was collected under aseptic conditions by a well-trained and authorized phlebotomist. Briefly, asepsis was done using ethanol at 70% and, using a needle of 21G, blood was collected by venipuncture at the bend of the elbow into heparin tubes. Bandage strap was used to avoid post-puncture infections. Collected blood samples were stored in coolers filled with ice packs and transported to the laboratory.

Once in the laboratory, malaria thick smears were prepared and analyzed as previously described by Ohrt et al. (40). Briefly, whole blood was mixed, and a spot placed in the center of a slide. Using the edge of another clean slide, red blood cells (RBC) were lysed to release *Plasmodium* parasites, and caution was taken to have a uniform thick smear (not too thin or too thick as they don't stain well). Prepared thick smears were air dry, stained with a freshly prepared Giemsa solution (10%) and analyzed using an optical microscope.

Subsequently, plasma samples were prepared by centrifugation and stored at −80°C until use. Hepatitis B and C diagnostics were performed using, respectively DiaSpot HBsAg and DiaSpot HCV Ab test strip from DIASPOT^TM^, Indonesia. Interleukin 33 levels was determined in plasma samples using Human IL-33 ELISA Kit from BioLegend, Inc. USA catalog number 435907. All the assays were performed following the instructions from the manufacturers.

### Statistical Analysis

Data were first entered in an excel sheet. Statistical analyses were conducted using R studio software and graphs were plotted using GraphPad Prism 6. Descriptive measures (means, medians, frequencies and percentages) were used to summarize data. Whereas, the *t*-test was used for comparison between two groups, the non-parametric Kruskal–Wallis test following by Dunn test were used for multiple comparison between more than two groups. Multiple Logistic regression was used to assess the risk of infection/liver pathology as a function of plasma IL-33 levels. Pearson r correlation was used to assess the correlation between the plasma level of IL-33 and the eggs burden. *p* < 0.05 were considered significant.

## Results

### Study Flow Diagram

**Figure 1:**
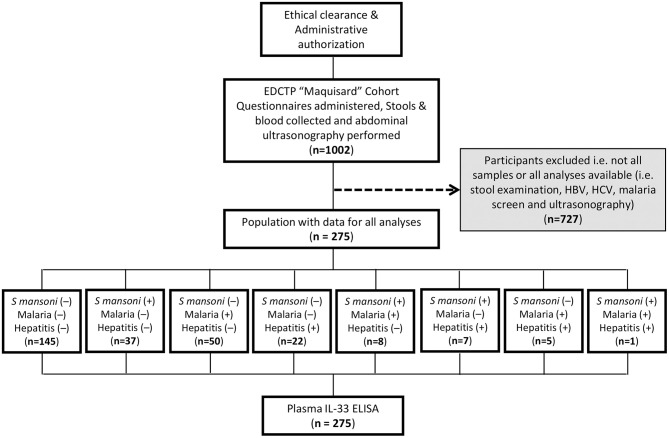
Study flow diagram describing the strategy of enrolment and examination of 275 schoolchildren from 5 villages from Bokito, Cameroon. Ethical and administrative authorization were secured to enroll 1002 school children from 5 public schools in rural Cameroon under the EDCTP-funded “Maquisard” cohort aimed at uncovering the host regulators of liver fibrosis in hepatic schistosomiasis. Recruited school children resided 1–14 years within the endemic area at the time of the enrolment. A battery of exams and diagnostic tests were performed on consenting participants and only samples from patients with no missing data for stool examination by Kato Katz, rapid diagnostic testing of Hepatitis B and C viruses and microscopical screening of malaria parasite in blood smears were further used in the present study. A total of 275 school children presented with all test results and were enrolled into the present sub-study. Their plasma samples were tested by ELISA for IL-33 quantification.

The participants and samples used for the present study were defined as indicated (Figure 1). Samples of participants missing any test result were excluded i.e., kato katz, malaria microscopy, hepatitis serology (B & C), or plasma IL-33. Finally, a total of 275 participants, age range of 6–16 years (mode of 10 years), with a male to female ratio of 1 (138/137) and a modal length of residence within the endemic area of 8 years (with a range of 1–14 years) were included.

### Characteristics of the Study Population

The participants' distribution by age and gender in the different groups of infections is presented below (Table 1).

**Table 1:** Age, gender, and infection status distribution of 275 schoolchildren from Bokito, Cameroon.

**Age groups**	**Study groups (N, %)**							
	**C**	**S**	**H**	**M**	**S + H**	**S + M**	**H + M**	**S + H + M**
	**(145, 52.7%)**	**(37, 13.5%)**	**(22, 8.0%)**	**(50, 18.2%)**	**(07, 2.5%)**	**(08, 2.9%)**	**(05, 1.8%)**	**(01, 0.4%)**
**<10 years**								
N (%)	33 (22.7)	10 (27.0)	05 (22.7)	17 (34.0)	00 (00)	02 (25.0)	02 (40.0)	00 (00)
M/F	8/25	1/9	3/2	10/7	0/0	0/2	0/0	0/0
**[10–14] years**								
N (%)	106 (73.1)	22 (59.5)	15 (68.2)	32 (64.0)	06 (85.7)	06 (75.0)	03 (60.0)	01 (100)
M/F	54/52	14/8	8/7	21/11	2/4	3/3	1/2	1/0
**>14 years**								
N (%)	06 (4.1)	05 (13.5)	02 (9.1)	01 (2.0)	01 (14.3)	00 (00)	00 (00)	00 (00)
M/F	3/3	5/0	2/0	1/0	1/0	0/0	0/0	0/0

*M, Male; F, Female; C, Control; S, Schistosomiasis; H, Hepatitis; M, Malaria; +, coinfection*.

The vulnerable age class of 10–14 was the most represented in our study population. Prevalences of 13.5% (37/275), 18.2% (50/275), and 8% (22/275) were found for schistosomiasis, malaria and hepatitis (B or C) single infections, respectively. *S. mansoni*-positive participants eggs burden ranged from 12 to 4,572 EPG (Eggs per gram of stool) with a geometric mean of intensity (GMI) of 186 EPG. Among them (*n* = 275) and including participants whose schistosomiasis status were singly known (*n* = 35), 83% (49/59), 11.9% (7/59), and 5.1% (3/59) had, respectively a light (EPG < 100), moderate (100 < EPG < 400) and heavy (400 < EPG) infection. In Malaria-positive participants, the parasitic load varied from 28 to 24,086 parasites per mm^3^ of blood with a mean of 453. A total of 7.6% (21/275) of the fully screened children presented with co-infection(s) of any sort (2.5% for schistosomiasis & hepatitis, 2.9% for schistosomiasis & malaria, 1.8% for malaria & Hepatitis, and 0.4% for schistosomiasis & hepatitis & malaria). Moreover, only 8 school children were found positives for other geohelminths (3 cases of *Trichuris trichiura*; 2 cases of *Ascaris lumbricoides*; 1 case of *Ancylostoma duodenale*; 1 case of *Ascaris lumbricoides & Ancylostoma duodenale* coinfection; *and 1* case of *Ascaris lumbricoides & Trichuris trichiura* coinfection). Finally, no ectopic elimination of *S. haematobium* eggs was recorded in participants stool.

### Schistosomiasis Infection Associates With Lower Plasma IL-33 Levels in a Polyparasitic Site

**Figure 2:**
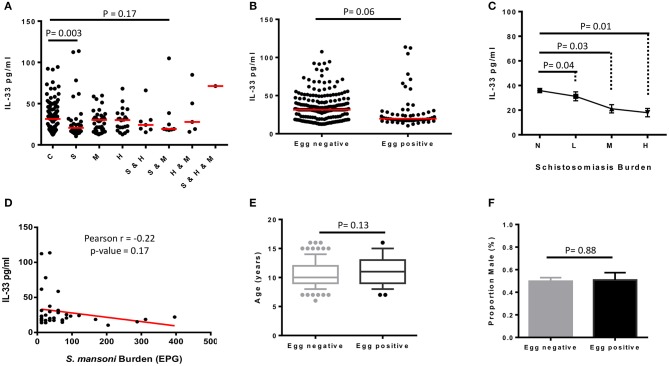
Plasma levels of IL-33 of schoolchildren from Bokito Cameroon, with Schistosomiasis (S); Malaria (M) and hepatitis B & C (H), single or coinfections, and in a control group (C) free of these infections. **(A)** Comparative graph of plasma level of IL-33 in individuals cataloged according to their schistosomiasis (S), malaria (M), and hepatitis (H) infectious status. Statistical analysis was performed using R to assess by Kruskal–Wallis test followed by Dunn test significant differences between groups. The horizontal bars represent the medians. **(B)** Plasma levels of IL-33 of all enrolled participants clustered uniquely according to their stool Kato katz result (*n* = 310) i.e., into *Schistosoma mansoni* egg negative and egg positive, irrespective of having a known parasitological status for malaria and/or hepatitis. Statistical comparison was performed by two-sided unpaired *t-*test using R software. The horizontal bars represent the medians. **(C)** All *S. mansoni* egg positive participants, including those with unknown malaria and/or hepatitis status, were clustered based on the number of eggs per gram (EPG) of stool into L: light (<100 EPG; *n* = 49), M: moderate (100–399 EPG; *n* = 7), H: heavy (>400 EPG; *n* = 3). *S. mansoni* negative (N; *n* = 251) participants, including those with unknown infection status for malaria and hepatitis. Plasma IL-33 values were plotted for each group of egg burden and comparison between groups were performed in a two-by-two approach by two-sided unpaired *t-*test. Values are displayed into means ± sem. **(D)** Correlation between the plasma level of IL-33 and *S. mansoni* eggs burden (EPG) of positive participants with known infection status for malaria and hepatitis was assessed by Pearson correlation using R software. Age **(E)** and sex **(F)** distribution of egg negative and egg positive participants were compared by two-sided unpaired *t-*test using R software. The boxplot representing age shows the median, the 1st and 3rd quantiles including outliers. The samples are represented to show the median age and mean proportion of males.

**Figure 3:**
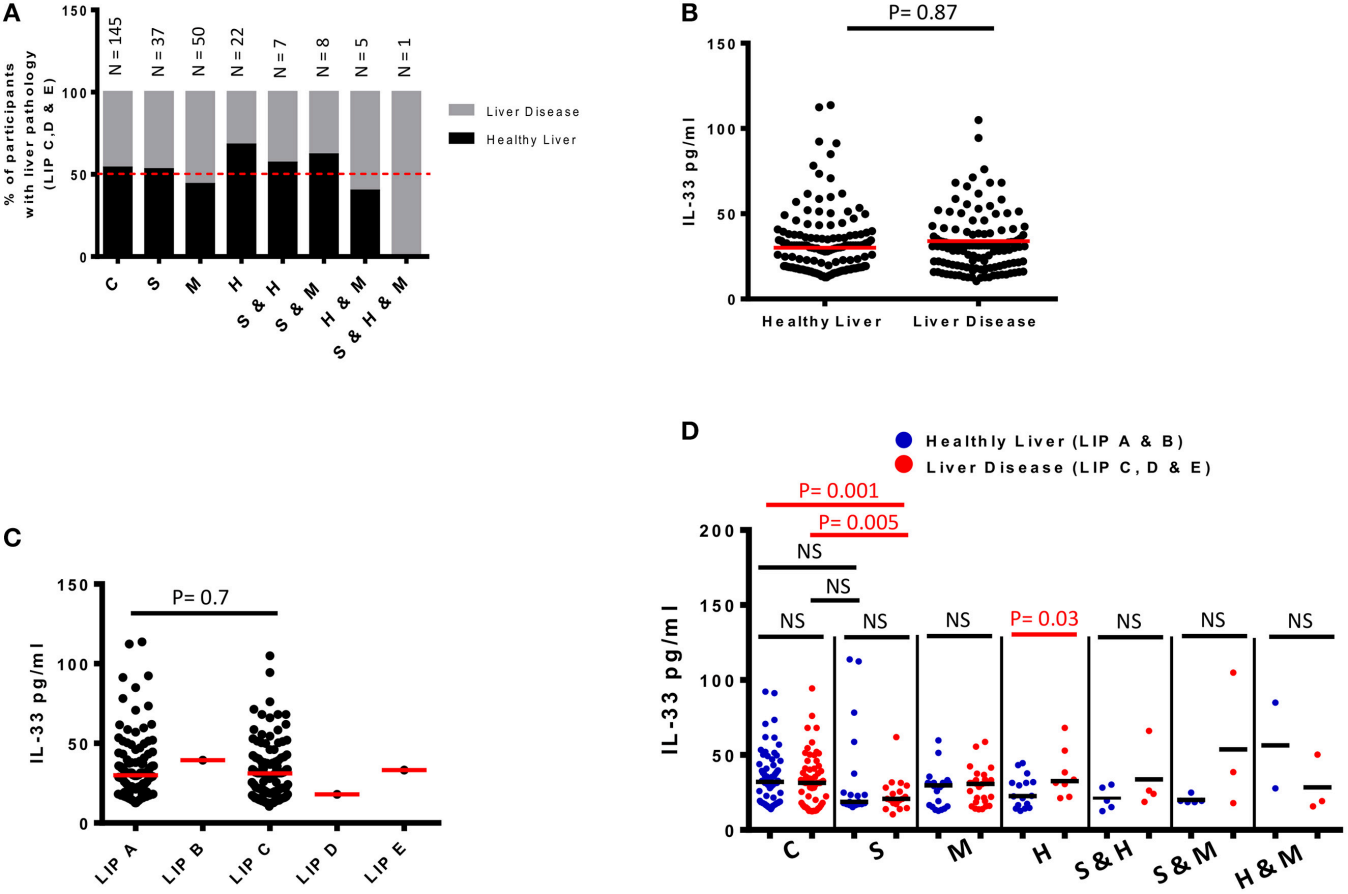
Plasma levels of IL-33 and infection-associated liver disease detected by ultrasound in 275 schoolchildren from Bokito Cameroon, with Schistosomiasis (S); Malaria (M) and hepatitis B & C (H), single or coinfections, and in a control group (C) free of these infections. **(A)** Proportions of participants with liver image pattern C, D & E when compared to total participants screened in each infectious category. An interrupted line is drawn at 50%. Total number of participants for each cluster is also indicated on top of the bars. **(B)** Plasma levels of IL-33 for participants with healthy liver image patterns (A & B) when compared to participants with pathological liver image patterns (C, D & E). The difference between the groups was assessed by unpaired *t*-test using R software. The red horizontal lines represent the medians. **(C)** Plasma levels of IL-33 for participants clustered according to each grade of liver image patterns. Differences are assessed by unpaired *t-*test using R software. The red horizontal lines represent the medians. **(D)** Plasma levels of IL-33 for participants per infectious status separated into those from participants with healthy LIP (A & B) vs. plasma IL-33 levels of participants from the same infectious cluster with liver disease (LIP C, D & E). Differences between plasma IL-33 levels of participants with and without liver disease from a given infectious cluster were assessed by two-tailed unpaired student *t*-test within the infectious cluster concerned. NS, not significant, *p* > 0.1. The black horizontal lines represent the medians.

To assess whether plasma levels of IL-33 were different from a disease to another, plasma IL-33 concentrations were represented for all participants according to their infection status as reported in Table 1 (Figure 2A). A minimal negative association was observed between plasma IL-33 levels and schistosomiasis infection (AOR 0.99; 95% CI 0.97–1.01). As shown in Figure 2, we further observed a significant reduction of plasma IL-33 levels in schistosomiasis only infected participants when compared to controls indicating a possible negative association of this cytokine with schistosomiasis infection (*p* = 0.003). Moreover, all participants with schistosomiasis, co-infected or not, appeared to have a lower plasma concentration of IL-33 as demonstrated by the comparison of schistosomiasis negative and schistosomiasis positive participants irrespective of their status relating to other diseases screened (Figure 2B). This was convincingly substantiated by the observation that plasma IL-33 levels decreased with egg burden in our study population (Figure 2C) clearly establishing the negative association between plasma IL-33 and schistosomiasis infection in school children from this polyparasitic area of rural Cameroon. A negative Pearson correlation coefficient (*r* = −0.22) further confirmed this observation (Figure 2D) despite the absence of mathematical significance (*p* = 0.17). Non-significant differences between age (Figure 2E) and gender distributions (Figure 2F) of egg-positive and egg-negative participants further reinforced the indication of a true negative association of schistosomiasis taken within a polyparasitic setting with plasma IL-33 concentration. Moreover, whereas the difference between controls without liver injury vs. schistosomiasis without liver disease was not significant (*p* = 0.55), schistosomiasis infected-participants with liver disease showed a significant lower plasma level of IL-33 (*p* = 0.005) when comparing to controls with liver injury (Figure 3D). In contrary, no difference of plasma IL-33 concentration was found between geohelminths-positive participants compared to geohelminths-negatives (*p* = 0.31) or compared to controls not infected with any of the tested pathogens (*p* = 0.29).

### Lack of Association Between IL-33 and Liver Disease in Schistosomiasis Endemic Area

Ultrasonography was used to assess liver disease in our study participants (Table 2, Figure 3) as per the WHO Niamey protocol for assessing liver disease in schistosomiasis endemic areas (31). Liver Image patterns (LIP) from A, B, C, D to E were observed in the screened school children (Table 2, Supplementary Figure 1). We observed liver image patterns (LIP) indicative of fibrotic pathology (grades C, D & E) in 47.3% (130/275) of our study population (Table 2) but participant distribution by LIP and disease cluster failed to reveal any preferential occurrence of liver disease (LIP grades C, D & E) in infected participants (schistosomiasis, malaria, or hepatitis) when compared to non-infected controls (63 infected vs. 67 non-infected participants with liver disease). Among participants infected with at least one of the screened infectious agents, liver fibrosis (Image patterns C, D, and E) was relatively more prevalent in cases of malaria (54.7% i.e., 35/64), followed by schistosomiasis (47.2% i.e., 25/53) then hepatitis (40% i.e., 14/35) (Figure 3B). Overall, LIP grade C was the most prevalent in participants with liver pathology (98.5% i.e., 128/130). No difference (*p* = 0.9) was found in the IL-33 plasma levels of participants with liver disease (LIP C, D, E) compared to controls with no signs of liver disease (LIP A, B) (Figure 3C). Moreover, comparison of plasma IL-33 levels between participants having different LIPs (LIP A vs. LIP B vs. LIP C vs. LIP D vs. LIP E) also failed to reveal a significant difference (*p* = 0.7) (Figure 3D). Disease-specific comparison of plasma IL-33 levels between participants with healthy (A & B) and pathological (C, D & E) liver image patterns (LIPs) revealed a significant elevation of IL-33 plasma levels only in hepatitis-positive participants with liver disease when compared to hepatitis-positive participants without liver disease (Figure 3D). No such difference in plasma IL-33 concentration could be observed in either malaria or schistosomiasis-diseased participants. Ultimately, this lack of association between plasma IL-33 levels and liver disease was further corroborated by multiple regression analysis (AOR 1.00; 95% CI 0.99–1.01).

**Table 2:** Liver Image patterns, liver disease proportion, gender and infection status of 275 schoolchildren from Bokito, Cameroon.

**Liver image pattern**	**Study groups (N, M/F)**							
	**C**	**S**	**H**	**M**	**S + H**	**S + M**	**H + M**	**S + H + M**
	**(145, 65/80)**	**(37, 20/17)**	**(22, 13/9)**	**(50, 32/18)**	**(07, 3/4)**	**(08, 3/5)**	**(05, 1/4)**	**(01, 1/0)**
LIP A (*N*, %)	77 (53.1)	19 (51.3)	15 (68.2)	22 (44.0)	04 (57.1)	05 (62.5)	02 (40.0)	00 (00)
LIP B (*N*, %)	01 (0.7)	00 (00)	00 (00)	00 (00)	00 (00)	00 (00)	00 (00)	00 (00)
LIP C (*N*, %)	67 (46.2)	17 (46.0)	07 (31.8)	27 (54.0)	03 (42.9)	03 (37.5)	03 (60.0)	01 (100)
LIP D (*N*, %)	00 (00)	01 (2.7)	00 (00)	00 (00)	00 (00)	00 (00)	00 (00)	00 (00)
LIP E (*N*, %)	00 (00)	00 (00)	00 (00)	01 (02)	00 (00)	00 (00)	00 (00)	00 (00)
LIP C + D + E (*N*, %)	67 (46.2)	18 (48.6)	7 (31.8)	28 (56.0)	3 (42.9)	3 (37.5)	03 (60)	1 (100)

*M, Male; F, Female; LIP, Liver Image Pattern; C, Control; S, Schistosomiasis; H, Hepatitis; M, Malaria; +, coinfection*.

## Discussion

The world health organization has encouraged research helping the monitoring of the effectiveness of intervention programs deployed against schistosomiasis. Increasingly, research has therefore focused on defining parameters that would refine the monitoring of treatment effectiveness in reducing the disease prevalence and associated morbidity in areas subjected to mass drug administration, the principal control tool of the global anti-schistosomiasis control/elimination strategy (42).

Infection status thus success of treatment has been popularly monitored by changes in stool/urine examination, blood serology, and PCR. Morbidity has been evaluated by hematological assays (for anemia), intellectual performance (for cognitive impairment) (43) and ultrasonography (for tissue destruction) (37). The indirect measurement of blood factors that dynamically respond to infection and disease progression are also being gradually evaluated (44, 45).

In our study, we evaluated the latter group of indicators by questioning the association of an important host alarmin, interleukin-33, with schistosomiasis infection and/or liver fibrosis in a well-characterized foci of schistosomiasis in rural Cameroon where other infectious diseases (malaria, hepatitis B, and Hepatitis C) are also common (4). Our major finding is that of a significant propensity of schistosomiasis-infected hosts to have lower plasma IL-33 levels when compared to non-infected hosts. Notably, the strongest reduction was observed in participants with the highest burden of *S. mansoni* eggs indicating a solid and hitherto unreported negative association between schistosomiasis infection and plasma IL-33 levels.

Our study first comprehensively investigated the burden of *S. mansoni, Plasmodium spp* and Hepatitis B and hepatitis C viruses in school children from five public schools of the polyparasitic site of Bokito in rural Cameroon (4). Only participants successfully screened for all diseases were included in the present study. Two hundred and seventy-five included participants, aged 6–16 years old were therefore all screened for schistosomiasis, malaria, hepatitis B, and hepatitis C. We reported positive cases for all diseases with overall prevalences ranging from 23, 19, to 13% for schistosomiasis egg positive cases, malaria positive microscopy to hepatitis serology positive cases, respectively. Two stool samples were collected from each participant and analyzed by Kato Katz (KK) by two independent and experienced technicians, since such a proceeding might improve the sensitivity of KK smears (46–48). Nevertheless, *S. mansoni* prevalence might be higher in our study population considering the overall low sensitivity of the Kato-Katz smear (49, 50). In the light of such a limitation, however, our findings might miss cases of low infection burden and shed a focused light on KK-detectable infections only. Notably, also, are the moderate to high levels of schistosomiasis infections in our study population that might suggest either a very swift rate of reinfection in our study area. In fact, evidences in favor of the high and rapid re-infection rates of *S. mansoni*, within a six-month period following treatment, have been previously reported on the site (4) to substantiate our interpretation. Nevertheless, limited adherence of these School-aged Children (SAC) to the MDA program 5 months ago could have also contributed to the prevalence and infection burden. A comparative site study on the determinants of reinfection/persistent infection should be investigated to provide more clarification to these possibilities. Although, defined as highly focal in the transmission of hepatosplenic schistosomiasis (4), our present site participants were not screened for urinary schistosomiasis and such undertakings should be integrated in the future to update the dynamics of schistosomiasis transmitting species on the site. As of now, the absence of ectopic *S. hematobium* infection in our study population sufficiently argues against the likelihood of *S. mansoni*/*S. hematobium* co-occurrence on this site.

What was clear is that *Plasmodium falciparum* was the most prevalent of the single infection on the study site, consistent with a similar recent study in Uganda (51). However, our malaria prevalence was far lower than those reported elsewhere (51, 52). This could be explained by the fact that, unlike our study, children below 5 years old, the age at higher risk for malaria (53), are usually the primary target of malaria studies on children. Notably for schistosomiasis, our presently reported prevalence of *S. mansoni* eggs in the participants' stools is lower than that recently reported in the region (4). A possible explanation is the difference in the geographical coverage of both studies where the previous only screened the two most affected public schools and the present study extended the enrolment to three more public schools in the sub-district of Bokito. Moreover, this low prevalence could be also explained as our study was conducted just 5 months after Mass drug administration (MDA), which could further explain the overall occurrence of a light burden in our study population (GMI: 35 EPG). This contrasts a study performed in Senegal where the overall burden was moderate (GMI: 120 EPG) (54). In contrast to previous studies (7, 8), hepatitis prevalence was quite low in our setting. This is certainly related to the age range of our study population which is considerably much younger than cohorts investigated in local hepatitis studies (7, 8).

Alarmingly, all scenarios of mixed infections were observed in our rather young and rural study population further reinforcing the need to approach infectious diseases research in affected tropical foci from a polyparasitic rather than monoparasitic angle of consideration.

### On the Changes of Plasma IL-33 Levels During Infection

*Schistosoma mansoni*-infected participants displayed lower levels of plasma IL-33 suggesting the possible depletion of plasma IL-33 during infection. This result, although striking, contrasts with other studies suggesting the accumulation of this factor during the inflammatory response that characterizes *S. japonicum* in humans and mice (55, 56). Such a difference could well be ascribed to the differential pathophysiology of *S. mansoni* and *S. japonicum* (57). Possibly, IL-33, secreted principally by the epithelium, fibroblasts and endothelium, released after cell damage caused by *S. mansoni* eggs will bind to its receptor (ST2) and drives the T helper 2 response (58, 59) needed to contain the infection. This binding might therefore reduce the plasma levels of IL-33 which is known to decay 2 h after production by inactivation in the extracellular environment through oxidation of cysteine residues and the establishment of two disulfide bonds in the IL-1-like cytokine domain (60). *S. mansoni* infections in our clinical settings are rather chronic by nature. Therefore, the acute profile of *S japonicum* infections in these previous studies argue for the differential availability of IL-33 in the plasma of subjects infected with *S. japonicum* or *S. mansoni*. A possible explanation of the observed low IL-33 plasma concentration in individuals chronically infected with *S. mansoni*, as is the case in our study, could be the early solicitation/utilization of this factor (IL-33). In fact, this line of thoughts gains support from a recent study that also reported low IL-33 blood levels in *S. mansoni* positive participants (41).

Another possible explanation for the reduced levels of plasma IL-33 in *S. mansoni* infected participants we observed could be the parasite-driven impairment of IL-33 production (41). Such a stratagem has recently been described for a parasitic nematode where a factor, HpARI (Alarmin release inhibitor) was identified and shown to tether IL-33 to necrotic cells preventing its release (61). Whether such an immunoregulatory process is also shared by *S. mansoni* should be investigated to address this. Additionally, a recent report showed that IL-33 is downregulated when its soluble receptor (sST2) is upregulated in the serum of patients with inflammatory bowel disease (62). A case is robustly made for the role of the soluble forms of ST2 might have as decoy receptors that neutralize IL-33 in biological fluids (63). Clearly, the assessment of this soluble form of the IL-33 receptor (sST2) could provide further indication on the mechanistic bases of the reduction of plasma IL-33 concentration in *S. mansoni* infected children.

As of now, the reduction of plasma IL-33, an innate lymphoid type 2 cell (ILC2) activating factor (64), in *S. mansoni*-infected children in our study parallels the reported reduction of ILC2 and TSLP in young children from rural Zimbabwe infected with *S. haematobium* (65). Although no differences in alarmin levels were noted in this previous study between. *S. haematobium* egg negative and egg positive participants, a likely explanation for the discrepancy with our study could be the limited sample size used per age groups to assess the differences [12 participants (65)]. Our study, with a more robust sample size (275 participants) reliably revealed the reduction in this alarmin in infected children that ultimately aligns with the reduction in ILC2 (performed by those authors with a larger sample size cumulating all age groups) reported in this previous study (65). In fact, the consolidation of our observation is made by these authors allusion to a significant effect of a specific IL-33 single nucleotide polymorphisms (SNP) showing that allele variation influences schistosome infection intensity (65).

No difference was found in the blood level of IL-33 among participants with other single infections failing to demonstrate a critical role of IL-33 in the plasma of individuals during malaria of hepatitis infections. However, reports have been made on the elevated levels of IL-33 in patients chronically affected by hepatitis B or C (31, 32) and on the Increased IL-33 levels in the plasma of patients with severe falciparum malaria with a protective role (66). These particularities could well be missing from our cohort which is poorly characterized with regards to malaria and hepatitis disease sub-groups (asymptomatic, advanced, severe, chronic…). Further studies are therefore needed with better characterized cohorts to conclusively address the role of IL-33 in these diseases (malaria and hepatitis).

### On IL-33 and Infection-Driven Liver Disease

Our study revealed that most participants with pathological liver image patterns (LIP) had a grade C. This is consistent with our previous study in the same sub-district (4) and a similar study in a schistosomiasis-endemic area conducted in Northern Senegal (54). LIP C readily refers to liver pathology ranging from possible to established liver fibrosis in schistosomiasis endemic areas (31). LIP D and E, which reliably denote established to advanced fibrotic pathology in the liver (31), were very few and no LIP F was recorded in contrast to the latter study in Northern Senegal. This might be explained by the fact that our participants were schoolchildren (age range from 6 to 16 years) in an area subjected at least once yearly to mass administration of praziquantel (4).

Participants with the highest proportion of liver disease and the worst liver profile (LIP E) were from Infections with *P. falciparum*. This raises the question of the involvement of Malaria in liver pathology in this polyparasitic setting. In fact, this is not uncommon as previous reports have readily identified the liver as a crucial organ for *Plasmodium*'s life cycle (9, 67) and pathological consequences of falciparum malaria within the liver have been reported (68). Remarkably, up to 46.2% (64/145) of liver pathology (LIP C) was also recorded in negative controls. This either (i) further reinforces the limited sensitivity of Kato Katz based screen of our participants where cases with low egg burden might have been missed or (ii) might come as a result of previous infections which were cleared following PZQ treatment, as our study was conducted just 5 months after general MDA in the study population. In fact, even though pathology amelioration follows treatment, the low reversal speed and the high likelihood of reinfection might make it difficult for some treated individuals to rapidly become pathology-free. Notably, however, no case of severe liver pathology (LIP D & E) was recorded in the control group in line with an overall limited progression of pathology following MDA (69, 70).

Intriguingly, participants with schistosomiasis tended to display a reduced proportion of liver disease when co-infected with malaria or hepatitis. In as much as the limited sample size of co-infected patients might limit the strength of this observation, this is not unprecedented. Mixed infections involving schistosomiasis have been increasingly reported to ameliorate the disease prognosis when compared to single schistosomiasis infections (54, 71, 72). Although the mechanistic insights of such an important dynamic of schistosomiasis-driven liver pathology in polyparasitic areas is still poorly understood, a possible explanation is the immune modulation of concomitant infections on the course of schistosomiasis [Th1 in the context of malaria and hepatitis (14, 73) and Tregs for other helminth infections]. This could mitigate the progression of the fibropathological reaction caused by single schistosomiasis infections. Dissimilar patholophysiological processes of these diseases might account for the antagonized disease progression in some co-infections. This is not the case in Malaria-Hepatitis coinfection cases, which displayed the highest proportion of liver disease. A possibility here could be the shared physiopathological processes between the 2 diseases whereby *P. falciparum* sporozoites were shown to enter hepatocytes using the same receptor as Hepatitis viruses (18). Clearly, these assertions on the differential pathology that ensue co-infections should be taken with caution considering the sample size of our coinfected groups. A broader study including adults would therefore be needed to efficiently assess the contribution of these coinfections on the modulation of the liver disease in schistosomiasis endemic areas.

Our data failed to show a significant difference in the plasma levels of IL-33 in participants with liver disease compared to healthy ones from most infection groups except hepatitis. An in-depth appraisal of the morbidity predictive value of plasma IL-33 during hepatitis should be pursued to validate this preliminary observation. Notably, however, though the plasma levels of IL-33 in *S. mansoni*-infected participants with liver disease was not different to those without liver disease, these levels were lower than those of the egg-negative control groups. Intriguingly, plasma IL-33 levels in *S. mansoni*-infected participants without liver disease were apparently lower than levels of plasma IL-33 in controls but did not achieve statistical significance. These IL-33 plasma levels in *S. mansoni*-infected participants without liver disease did not differ from those of *S. mansoni*-infected participants with liver disease, as well but showed no apparent reduction when compared to the latter. A notable reason for this singularity of plasma IL-33 levels from participants with *S. mansoni* infection only without liver disease is their high dispersion (mean ± SD of 32.58 ± 30.24 pg/ml when compared to 23.22 ± 11.17 pg/ml for plasma IL-33 levels of *S. mansoni*-infected children with liver disease). Although, the reason for this high dispersion remain unknown to us, the mean values of IL-33 plasma levels in *S. mansoni*-infected participants of our cohort are consistently lower than that of control participants. This further confirms our claim of a reduced plasma level of IL-33 in the context of *S. mansoni* infection. As of yet, our attempt to define the clinical value of IL-33 in monitoring schistosomiasis-driven liver disease in school children from a polyparasitic site does not support the use of this clinical parameter. This indicated a poor predictive value for plasma IL-33 in assisting the monitoring of liver disease progression in schistosomiasis-diseased school children in our cohort from a polyparasitic area. Our overall observation of a lack of association between plasma IL-33 and schistosomiasis-driven live disease progression is contrasting with previous reports that have suggested the increase of IL-33 with hepatic fibrosis in general (29, 74) and hepatic schistosomiasis-driven liver fibrosis in particular (56). It should however be noted that our study singly investigated the plasma levels of the cytokine as a potentially less invasive approach to monitor pathology whereas some of these studies reporting the elevation of IL-33 as a result of hepatic fibrosis assessed the level of the cytokine within the liver tissue (29). Our data might therefore reconcile with an increase of IL-33 in the fibrotic liver and not the blood to associate/mediate the progression of liver fibrosis. In fact, this possibility is consistent with mechanistic studies in murine models which showed that IL-33 driven liver fibrosis is through the expansion of liver resident innate lymphoid (ILC2) (75) or through induction of M2 macrophages in liver tissues (56), confirming the principal requirement of IL-33 in the liver tissue.

In conclusion, our study in the sub-district of Bokito in rural Cameroon shows that plasma IL-33 levels were lower in children with Schistosomiasis independent of co-infection with malaria, hepatitis B or C or geohelminths. No change could be observed in children plasma IL-33 levels when liver disease was present or not arguing against the association of liver disease with IL-33 in our cohort. Therefore, future studies using a comparative high throughput screen of factors differentially expressed by schistosomiasis-diseased subjects presenting liver disease to those with healthy liver to comprehensively identify host morbidity markers during schistosomiasis is desperately needed.

## Data Availability Statement

All datasets generated for this study are included in the article/Supplementary Material.

## Ethics Statement

Ethical approval was obtained from the Cameroon National Ethics committee for Human Health Research (Approval No. 2018/02/976/CE/NECRHH/SP) followed by authorisations from the Ministry of Basic Education and Ministry of Public Health the of Cameroon (631-12.18). Written informed consents were obtained from all participants and all research was performed in accordance with the relevant guidelines/regulations. Local authorities and schools directors were also informed and granted us with authorizations. Assisted by school teachers, children and legal guardians were informed of the scope of the study. Written informed consents and assents were given by children and legal guardians. All data were treated anonymously by the research team. All Schoolchildren enrolled were treated with Praziquantel regardless of their parasitological status.

## Author Contributions

JN and SK: conceptualization, data curation and data analysis, formal analysis, and writing—original draft. JN and FB: funding acquisition. SK, FK, EK, LM, GT, FN, YO, MT, AO, MH, PN, RG, MK, and JN: investigation. JN, SK, FK, EK, LM, RG, MK, and FB: methodology. JN, SK, EK, LM, RG, MK, and FB: project administration. JN, RG, MN, MO, and FB: resources. JN, FB, RG, MK, and BO: supervision. JN, SK, FK, EK, PN, RG, and MO: writing—review and editing.

### Conflict of Interest

GT is employed by CIAB EXACT Medical Laboratory and MN is employed by LABOREB Cameroon. The remaining authors declare that the research was conducted in the absence of any commercial or financial relationships that could be construed as a potential conflict of interest.

## Supplementary Material

Supplementary Figure 1 Representative ultrasonograms from some of the 275 schoolchildren from Bokito Cameroon. Liver image patterns A, B, C, D, & E are displayed as assessed by the radiologist. **(A)** LIPA shows the representation of a normal liver. **(B)** LIPB illustrates starry sky which are echogenic foci within the liver parenchyma. **(C)** LIPC displays pipe stems which are echogenic ring diffuse within the liver parenchyma. **(D)** LIPD displays echogenic ruff around the portal bifurcation and **(E)** LIPE is the representation of highly echogenic patches from the portal vein to the parenchyma.
