# Supplementary figures and images for: Negative Association of Interleukin-33 Plasma Levels and Schistosomiasis Infection in a Site of Polyparasitism in Rural Cameroon
Source: Front Immunol. 2019 Dec 3;10:2827. doi: 10.3389/fimmu.2019.02827 (PMC6901687; doi:10.3389/fimmu.2019.02827)

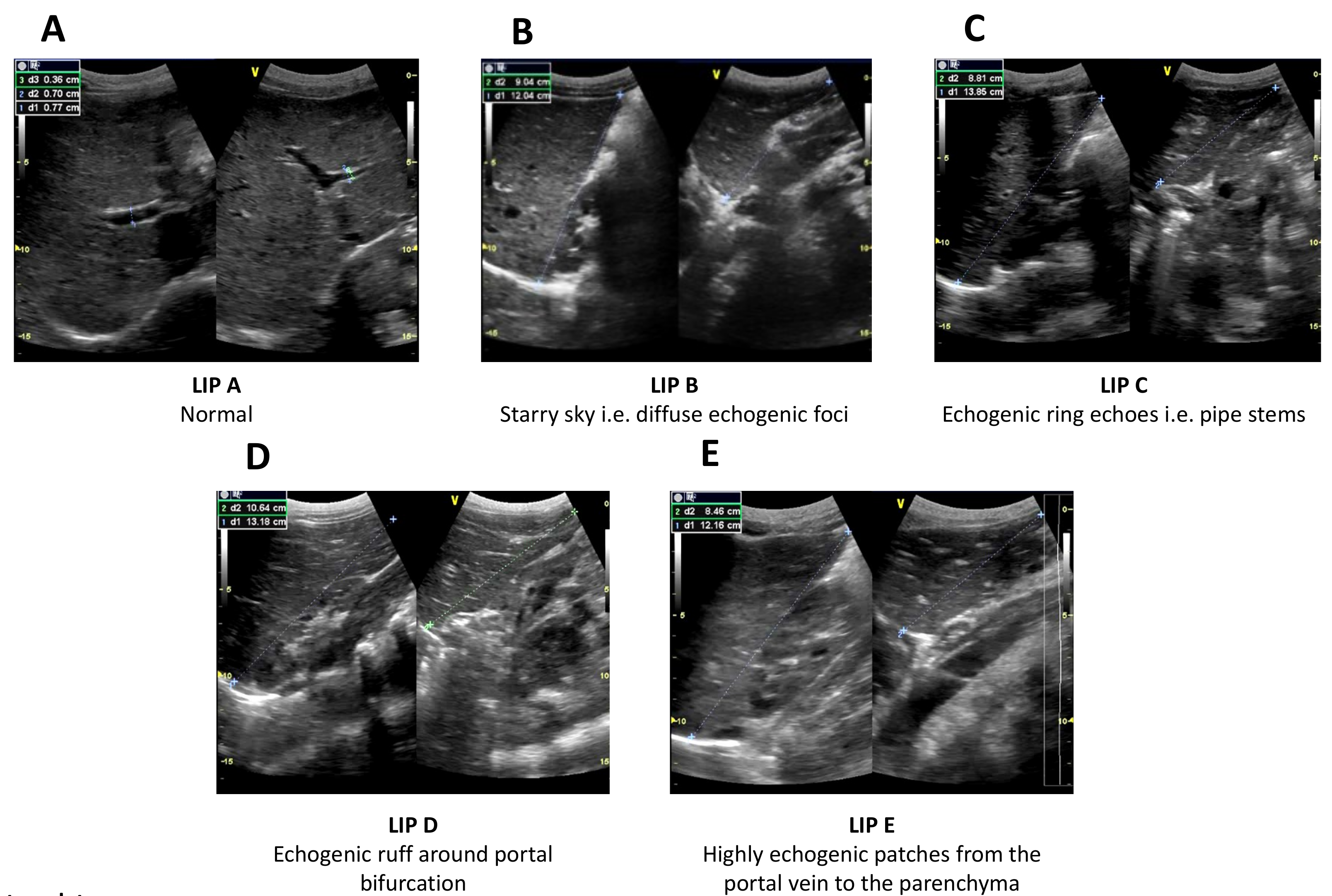

Supplement: Supplementary Figure 1 — Representative ultrasonograms from some of the 275 schoolchildren from Bokito Cameroon. Liver image patterns A, B, C, D, & E are displayed as assessed by the radiologist. (A) LIPA shows the representation of a normal liver. (B) LIPB illustrates starry sky which are echogenic foci within the liver parenchyma. (C) LIPC displays pipe stems which are echogenic ring diffuse within the liver parenchyma. (D) LIPD displays echogenic ruff around the portal bifurcation and (E) LIPE is the representation of highly echogenic patches from the portal vein to the parenchyma. [file Image_1.TIF]
